# Dose Profile Modulation of Proton Minibeam for Clinical Application

**DOI:** 10.3390/cancers14122888

**Published:** 2022-06-11

**Authors:** Myeongsoo Kim, Ui-Jung Hwang, Kyeongyun Park, Dohyeon Kim, Hak Soo Kim, Sang Hyoun Choi, Jong Hwi Jeong, Dongho Shin, Se Byeong Lee, Joo-Young Kim, Tae Hyun Kim, Hye Jung Baek, Hojin Kim, Kihwan Kim, Sang Soo Kim, Young Kyung Lim

**Affiliations:** 1 Department of Radiation Oncology, Proton Therapy Center, National Cancer Center, Goyang 10408, Korea; 95299@ncc.re.kr (M.K.); kypark@ncc.re.kr (K.P.); kimdoo20@ncc.re.kr (D.K.); haksoo.kim@ncc.re.kr (H.S.K.); jonghwi@ncc.re.kr (J.H.J.); dongho@ncc.re.kr (D.S.); sblee@ncc.re.kr (S.B.L.); jooyoungcasa@ncc.re.kr (J.-Y.K.); k2onco@ncc.re.kr (T.H.K.); 2 Department of Radiation Oncology, College of Medicine, Chungnam National University, Daejeon 35015, Korea; uijunghwang@cnu.ac.kr (U.-J.H.); khkim@cnuh.co.kr (K.K.); 3 Department of Radiation Oncology, Korea Cancer Center Hospital, Seoul 01812, Korea; shchoi@kirams.re.kr; 4 Radiological Science Branch, Research Institute, National Cancer Center, Goyang 10408, Korea; hjb@ncc.re.kr; 5 Department of Radiation Oncology, Yonsei Cancer Center, Yonsei University College of Medicine, Seoul 03722, Korea; hjhenrykim@yuhs.ac

**Keywords:** spatially fractionated radiation therapy, proton therapy, proton minibeam radiation therapy, multislit collimator, scatterer, peak-to-valley dose ratio

## Abstract

**Simple Summary:**

Proton minibeam radiation therapy (pMBRT) using multislit collimator (MSC) and scatterers has been proposed to spare healthy tissues and organs on the beam path and beyond the Bragg peak. An MSC that was much thicker than the maximum range of the proton beam could provide a sufficiently high peak-to-valley dose ratio at the patient’s skin, and the scatterers could actively convert the spatially fractionated proton beam to a uniform and broad beam in tumors by changing their thickness. The combination of the MSC and the scatterers can be a good solution for implementing pMBRT in clinical proton therapy facilities.

**Abstract:**

The feasibility of proton minibeam radiation therapy (pMBRT) using a multislit collimator (MSC) and a scattering device was evaluated for clinical use at a clinical proton therapy facility. We fabricated, through Monte Carlo (MC) simulations, not only an MSC with a high peak-to-valley dose ratio (PVDR) at the entrance of the proton beam, to prevent radiation toxicity, but also a scattering device to modulate the PVDR in depth. The slit width and center-to-center distance of the diverging MSC were 2.5 mm and 5.0 mm at the large end, respectively, and its thickness and available field size were 100 mm and 76 × 77.5 mm^2^, respectively. Spatially fractionated dose distributions were measured at various depths using radiochromic EBT3 films and also tested on bacterial cells. MC simulation showed that the thicker the MSC, the higher the PVDR at the phantom surface. Dosimetric evaluations showed that lateral dose profiles varied according to the scatterer’s thickness, and the depths satisfying PVDR = 1.1 moved toward the surface as their thickness increased. The response of the bacterial cells to the proton minibeams’ depth was also established, in a manner similar to the dosimetric pattern. Conclusively, these results strongly suggest that pMBRT can be implemented in clinical centers by using MSC and scatterers.

## 1. Introduction

Proton therapy has a dosimetric advantage over conventional X-ray therapy because of its unique depth dose profile, known as the Bragg peak [1,2]. This peak enables radiation oncologists to spare the normal tissues and organs behind the target volume. However, proximal tissue sparing is also necessary to achieve a more ideal proton therapy. Proton minibeam radiation therapy (pMBRT) [3,4,5,6,7,8,9,10] is a novel approach that spares the normal tissues and organs found along the path of the proton beam to the tumor by spatial fractionation, while maintaining tumor control through a homogeneous tumor dose similar to conventional proton therapy. Normal tissue sparing may be explained by the dose-volume effect [11,12], cell-signaling effect [13], or microscopic prompt tissue-repair effect, which leads to a rapid repair of vascular damage [14,15,16,17].

The homogeneous dose distribution inside the tumor can be achieved by the proton beam spread due to inherent beam divergence and multiple Coulomb scattering in the traversed tissue [4,18]. This mechanism may work properly for deep-seated small tumors, but not for shallow-seated tumors or large tumors that extend close to the skin, because of insufficient beam widening at shallow depths. Therefore, achieving dose homogeneity over the entire tumor can be a challenge [19] when maintaining a high peak-to-valley dose ratio (PVDR) in the skin, and a beam modulation technique is required to obtain a homogeneous tumor dose for every tumor. 

Recently, magnetically focused beam scanning techniques [19,20,21,22] or dynamic beam collimating technique for scanning beams [23] have been proposed to achieve a patient-specific beam modulation. These techniques have the advantage of being able to control minibeam size and spacing. However, the former techniques require major modifications of the existing proton beam nozzles or even the potential installation of a linear accelerator, and the latter has technical difficulties in synchronizing proton beams with high scanning speeds. Although many approaches have been proposed to implement pMBRT, it remains challenging to uniformly control the tumor dose.

In this study, a new method that could greatly improve the dose uniformity over the entire tumor volume in pMBRT was proposed and demonstrated. This method uses a pMBRT system that combines a multislit collimator (MSC) and a scattering device composed of multiple scatterers. The scatterers installed downstream of the MSC can increase the transverse momentum of the spatially fractionated proton beams, and then solve the problem of the dose heterogeneity inside the tumor for clinical application [19]. In our proposal, the MSC can maintain a high PVDR at the incident region of the proton beam, while the scattering device can actively control the dose homogeneity inside the tumor regardless of its depth by changing the thickness of the scatterer.

## 2. Materials and Methods

### 2.1. Monte Carlo Simulation

Monte Carlo (MC) simulations of proton minibeams generated by an MSC and a scattering device were performed to evaluate their dosimetric properties and to design them properly. For the MC simulations, the GEANT4-based TOPAS (v3.1.p3) simulation toolkit was used, and a virtual proton beam source was modeled based on pencil beam data measured on the Proteus-235 proton therapy system (IBA, Louvain-la-Neuve, Belgium) installed at the National Cancer Center in Korea. To validate the source model, lateral dose profiles of single-spot beams and spread-out Bragg peaks (SOBPs) were compared in water with those of a treatment planning system (TPS) (Eclipse version 13.7, Varian Medical Systems, Palo Alto, CA, USA), which has already been commissioned for clinical use. 

In this study, the PVDR was used as a measure of normal tissue sparing or tumor control in pMBRT. The PVDR at a depth was calculated using the equation $\frac{{\bar{D}}_{peak}}{{\bar{D}}_{valley}}$, where ${\bar{D}}_{peak}$ and ${\bar{D}}_{peak}$ represent the mean peak dose and mean valley dose, respectively, in the lateral dose profile at depth. All the peak doses inside the beam field were averaged to calculate the mean peak dose, as were the valley doses.

The variation in PVDR with depth was simulated for various leaf widths and center-to-center (c-t-c) distances of the MSC; the details of the procedure are described in the Supplementary Materials. The lateral dose profiles were compared by changing the thickness of the MSC from 5 cm to 10 cm in increments of 1 cm under the determined leaf width and c-t-c distance conditions. The dose profiles for parallel and diverging MSCs were also compared for the determined MSC thickness (i.e., 10 cm). The effect of the scattering plates on the lateral dose profile of the proton minibeams generated by the MSC along the water depth was investigated. The scattering plates were lead sheets, and their thicknesses were in the range of 0.25–3.75 mm.

### 2.2. Fabrication of Components for pMBRT

**Figure 1:**
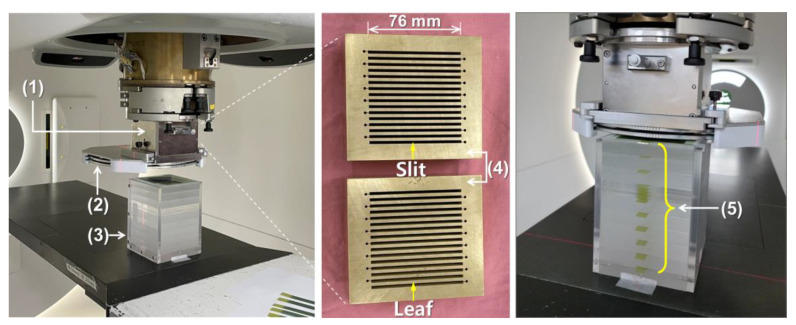
Experimental setup for dosimetry of proton minibeams: (1) an multislit collimator (MSC) housing, (2) a scattering device, (3) a polymethylmethacrylate (PMMA) phantom, (4) first and second 5 cm thick MSCs, (5) EBT3 film strips inserted at every 2 cm depth (except for one depth section) in the PMMA phantom that are perpendicular to the proton beam direction.

The MSC and scattering device designed using the MC simulation were fabricated (Figure 1). The MSC was made of brass (Cu 57.3%, Zn 38.8%) and consisted of a couple of MSCs with a thickness of 5 cm each for easy handling. The total thickness of each MSC was 10 cm, and their gap was 2 mm. Each smaller MSC had 16 slits, with a shape diverging from a virtual source point, and each slit in the second MSC was 2.5 mm wide and 76 mm long at the large end ((4) in Figure 1). In the scattering device, double-layered lead sheets were mounted as a scatterer and installed downstream of the MSC ((2) in Figure 1). The total thickness of the scatterer depends on the combination of the two lead sheets.

### 2.3. Measurement of Lateral Dose Profiles

Two broad proton beams with different SOBPs were designed in the Varian TPS using single-field uniform dose (SFUD) optimization and then delivered to a phantom using the pencil beam scanning (PBS) technique. The broad beams could deliver a dose of 4 CGE to the target in the absence of MSC. The range of the proton beams was 18.3 cm in water, which was comparable to the size of the patient’s head. The lateral dose profiles were measured using Gafchromic EBT3 films for the proton beams with SOBPs of 7 cm and 10 cm, respectively, and a field size of 60 × 60 mm^2^. To measure the profiles at various depths, a customized solid phantom with 2 cm thick polymethylmethacrylate (PMMA) slabs was fabricated to place the EBT3 films at appropriate depths ((3) and (5) in Figure 1). Proton minibeams were delivered under the experimental conditions of different scatterer thicknesses and different air gaps between the scatterer and the phantom. The irradiated films were scanned using a flatbed scanner (Expression 11000XL, Epson America Inc., Long Beach, CA, USA) with a scanning resolution of 200 dpi and analyzed using commercial software (RIT Classic, Radiological Imaging Technology, Colorado Springs, CO, USA). The EBT3 films in the same batch were used for the measurements, and dose calibration was performed before analysis.

The EBT3 films have the quenching effect, depending on the linear energy transfer (LET) in proton beams [24]. However, we did not take into account the quenching effect, because the proton pencil beams were delivered using SFUD technique and only relative doses were compared at every depth. The films were sandwiched between 2 cm thick PMMA slabs, and the farthest valid measurement position was 14 cm in depth, which was moderately far from the depth with pristine Bragg peak (range in PMMA = 16.2 cm). Even if the quenching effect exists there, it will be weak, and the same is true for peaks and valleys since the PVDR value is conceptually close to 1 around the pristine Bragg peak. 

### 2.4. Biological Response to Proton Minibeams

Ampicillin-resistant Escherichia coli cells (DH5α strain) were incubated in Luria-Bertani (LB) liquid medium with ampicillin at 37 °C overnight in a shaking incubator. Then, 3 × 10^8^ bacterial cells were mixed with 4 mL of pre-melted LB media containing 1% agarose and plated in a 100 mm petri dish. After hardening the LB agar plates, the plates were placed at different depths (surface, 2 cm, mid-SOBP) in the PMMA phantom and irradiated with 48 CGE at mid-SOBP. After proton minibeam irradiation, the plates were incubated at 37 °C for 16 h to assess for bacterial survival.

## 3. Results

### 3.1. Monte Carlo Simulation

#### 3.1.1. Validation of MC Simulation

**Figure 2:**
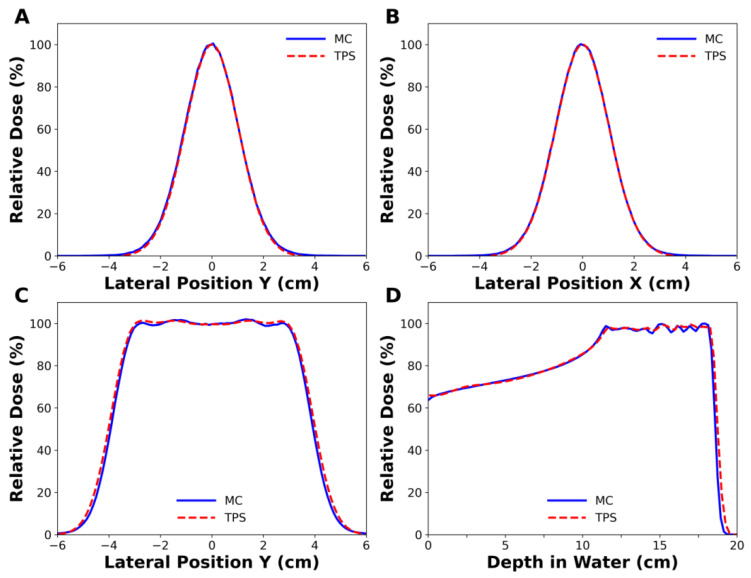
Comparison of the simulated dose profiles with those of treatment planning system (TPS). (**A**,**B**) belong to a single spot beam with 102.7 MeV energy, while (**C**,**D**) belong to spread-out Bragg peak (SOBP) beam with 163.3 MeV nominal energy. (**A**) 1σ in in-plane was 1.049 cm in Monte Carlo (MC) simulation and 1.048 cm in the TPS, and (**B**) 1σ in cross-plane was 1.050 cm in MC simulation and 1.062 cm in the TPS.

Figure 2A,B show the lateral dose profiles of a single-spot beam at the beam entrance obtained from the MC simulation and TPS. One sigma (σ) of the beam in the x-direction was 1.05 cm in the MC simulation and 1.062 cm in the TPS, with a difference of 1.1%. In the same manner, the difference of one sigma in the y-direction was less than 0.1%. For all pencil beam energies, the average beam size differences between them were 1.7% and 0.1% in the x- and y-directions, respectively. In Figure 2C,D, the lateral dose profiles and depth dose profiles of the SOBPs were compared between the MC simulation and TPS. Their lateral penumbras, defined by the lateral distance between the points having 20% to 80% of the maximum dose, were 0.96 cm and 1.01 cm, respectively, and the difference was 0.05 cm, corresponding to a discrepancy of 5.2%. Their widths of SOBPs, corresponding to a longitudinal distance between 90% dose points on the central beam axis, were 7.57 cm and 7.64 cm, respectively, and the difference was 0.07 cm, corresponding to a discrepancy of 0.9%. Similar agreements between the MC simulation and TPS were also obtained for higher-energy proton beams.

#### 3.1.2. Effect of a Diverging Slit on the Lateral Dose Profiles

**Figure 3:**
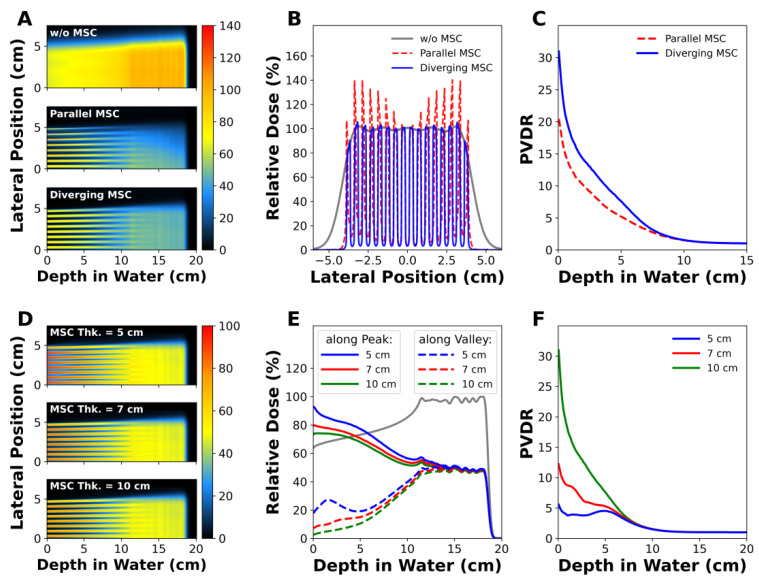
MC simulations of proton minibeams: (**A**–**C**) show the depth dose distributions, lateral dose profiles at the phantom surface, and peak-to-valley dose ratio (PVDR) profiles, respectively, of proton minibeams generated by parallel or diverging MSC. (**D**–**F**) show the influence of MSC’s thickness on the depth dose distributions, longitudinal dose profiles, and PVDR profiles in the diverging MSC.

Figure 3 shows the dose distributions that were simulated for the two types of MSCs with parallel or diverging slits. The flatness of the envelope covering the peak dose was 15.4% for parallel slits and 2.8% for diverging slits at the phantom surface (Figure 3B). The flatness is defined by $\left(D_{pmax}-D_{pmin}\right)/\left(D_{pmax}+D_{pmin}\right)\times 100$, where $D_{pmax}$ and $D_{pmin}$ are maximum and minimum doses among the peak doses in the field size, respectively. The MSC with diverging slits had a significantly higher PVDR than the MSC with parallel slits in the depth region between the beam entrance and the proximal SOBP (Figure 3C). Because this will appear more apparently in the large radiation fields, the use of parallel MSC will not be appropriate. Therefore, MSC with diverging slits are necessary for pMBRT to obtain a uniform dose distribution over the target volume. The diverging MSC was used in all experiments in this study. 

#### 3.1.3. Effect of MSC Thickness on PVDR

Figure 3D–F show the influence of the MSC thickness on the dose distributions and PVDR. According to the depth dose profiles along the peak and valley regions, the peak and valley doses decreased as the MSC thickness increased (Figure 3E). The PVDR at the phantom surface was 5.56 for the 5 cm thick MSC, increased to 12.2 for the 7 cm thick MSC, and reached up to 30.96 for the 10 cm thick MSC. The dramatic increase in PVDR for thicker MSCs was mainly due to the lower valley doses. For example, the valley dose for the 5 cm thick MSC was 17.74% at the surface, and it drastically decreased to 2.6% for the 10 cm thick MSC. Regardless of the MSC thickness, PVDR converged to 1 at depths comparable to the nominal beam range.

#### 3.1.4. Effect of the Scatterer on PVDR

**Figure 4:**
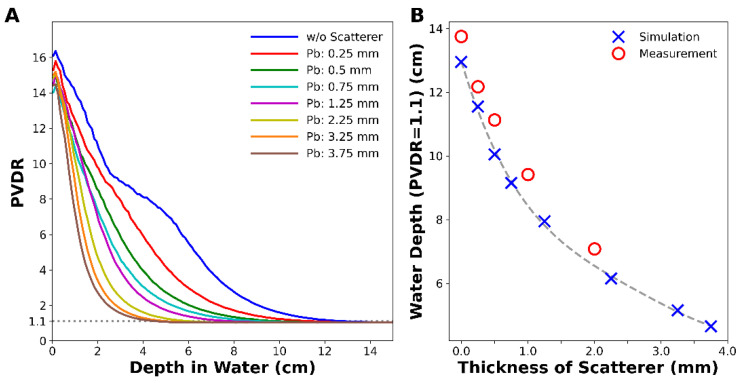
MC simulation of (**A**) PVDR with depth for various thicknesses of lead scatterer and (**B**) depth at which the PVDR became 1.1. Measured data were corrected by considering water-equivalent thickness of PMMA.

Figure 4 shows the changes in PVDR with depth for various thicknesses of the lead scatterer. The scatterer lowered the PVDR at depth, and the lowering power was proportional to the thickness of the scatterer without changing PVDR much at the surface (Figure 4A). As the scatterer became thicker, the water depth that satisfied the condition PVDR = 1.1, corresponding to a dose variation from 95% to 105%, became shallower. For example, the water depth satisfying PVDR = 1.1 was 12.9 cm without a scatterer, and it drastically decreased to 4.6 cm when a 3.75 mm thick lead scatterer was adopted (Figure 4B). This implies that a uniform dose can be delivered to a tumor by adjusting the thickness of the scatterer.

### 3.2. Measurement of Lateral and Longitudinal Dose Profiles

**Figure 5:**
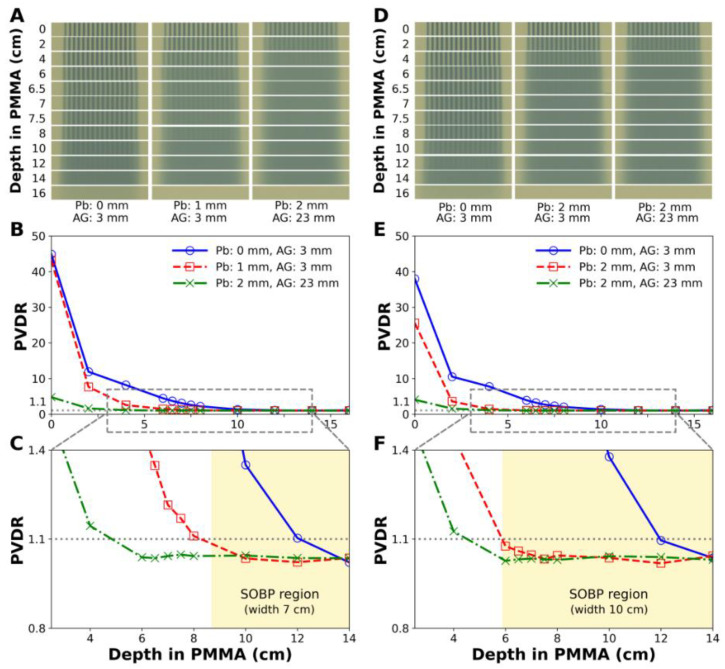
Dose distributions and PVDR measured by EBT3 films for proton minibeam with nominal energy of 163.3 MeV generated by a diverging MSC and a scattering device. (**A**–**C**) are for a 7 cm SOBP beam, and (**D**–**F**) are for a 10 cm SOBP beam. (**C**,**F**) are the magnified images of (**B**,**E**), respectively. The shaded areas indicate the SOBP regions. Pb: lead sheet, AG: air gap.

Figure 5 shows the lateral dose distributions and PVDR measured at various depths with the EBT3 films. In particular, Figure 5A,D show photographs of the film strips irradiated by the proton minibeams with 7 cm and 10 cm SOBPs, respectively, and the same nominal energy. Spatially fractionated and uniform dose distributions were observed together, and the uniform dose region expanded toward the phantom surface as the thickness of the scatterer increased. From the irradiated films, the PVDR could be calculated with depth.

For the 7 cm SOBP beam, the depth with PVDR = 1.1 shifted from 11.9 cm to 8.3 cm when a 1.0 mm thick lead scatterer was inserted (Figure 5B,C), resulting in uniform dose delivery throughout the SOBP. Similarly, for the 10 cm SOBP beam, the depth with PVDR = 1.1 shifted from 12.0 cm to 5.9 cm when a 2.0 mm thick lead scatterer was inserted (Figure 5E,F), resulting in uniform dose delivery throughout the SOBP again. When the air gap increased by 20 mm, the depth at which the PVDR became 1.1 decreased from 5.9 cm to 4.5 cm.

**Figure 6:**
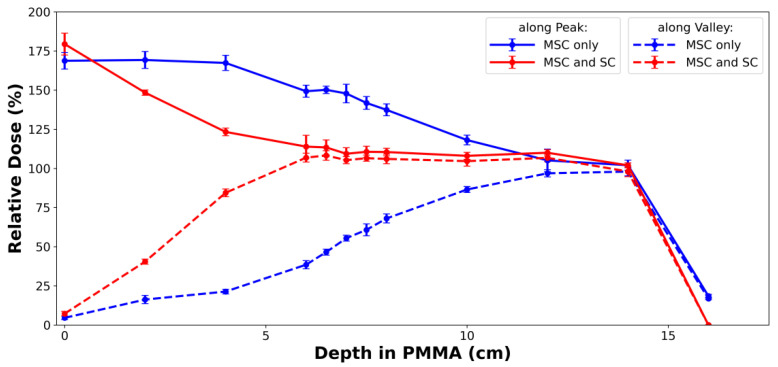
Comparison of depth dose profiles measured by EBT3 films (Figure 5D) for proton beams with 163.3 MeV nominal energy and 10 cm SOBP. The red-colored profiles show the effect of 2 mm thick lead scatterer. If the MSC is only used without the scatterer in pMBRT, a uniform dose cannot be delivered to the entire tumor volume. The measured doses were normalized by the most distal film dose, and there seemed to be weak quenching effect. The range of 163.3 MeV proton beam was about 16.2 cm in PMMA. SC: lead scatterer.

Figure 6 shows the depth dose profiles measured with the EBT3 films (Figure 5D). Looking at the valley dose curve, the proton beam passing through the MSC and the lead scatterer produced a homogeneous dose distribution over the 10 cm SOBP, whereas the proton beam passing through only the MSC did not. The high dose heterogeneity, i.e. high PVDR in the proximal region of the SOBP would be dominantly caused by the highest-energy proton beam, and the heterogeneity could not be eliminated by the lower-energy proton beams themselves. After passing through a 2 mm thick lead scatterer, the range of the proton minibeam was slightly shortened. There seemed to be a weak quenching effect of the EBT3 film at the most distal measurement position.

### 3.3. Biological Response to Proton Minibeams

**Figure 7:**
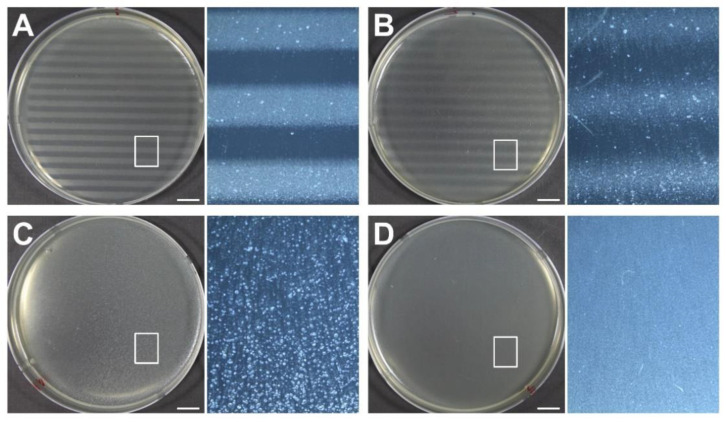
Biological responses of bacterial cells to proton minibeams at the various phantom depths, such as (**A**) surface, (**B**) 2 cm, and (**C**) mid-SOBP, and their comparison with (**D**) non-irradiated control cells. The doses delivered to the peaks and valleys were estimated to be 72 CGE and 2.5 CGE at surface, 72.3 CGE and 5.4 CGE at 2 cm depth, and 48.5 CGE and 47.8 CGE at mid-SOBP, respectively. Each panel at the right side shows magnification of the boxed area in the adjacent panel. Scale bars indicate 1 cm.

To determine the biological effects of proton minibeams, we irradiated the bacterial cells at the surface, at 2 cm depth, and at mid-SOBP, and subsequently examined cell survival. As shown in Figure 7, repetitive strip lines of Petri dishes, indicating high contrast of cell survival, can be seen at the surface (Figure 7A) and at 2 cm depth (Figure 7B) after irradiation with minibeams. A white milky pattern, indicating the homogeneous distribution of bacterial cells (Figure 7C), was detected in the irradiated cells at the mid-SOBP position as a non-irradiated control (Figure 7D). In the surviving population after irradiation, less damaged areas had a high number of surviving cells, small colonies, and milky patterns, whereas damaged regions retained a small number of surviving cells, appearing as a dotted pattern with large colonies. As shown in the microscopic images of the Petri dish located at the surface, the bright lines consisted of tiny dots of surviving colonies, while dark lines were clear, indicating that most of the cells were damaged and destroyed (right panel of Figure 7A). In contrast, the plate at the mid-SOBP showed that the alternative pattern was not identified, and their colonies looked larger and fewer (right panel of Figure 7C). The plate at 2 cm depth showed the smearing boundary and intermediate style of colony pattern between the surface and mid-SOBP (right panel of Figure 7C). Taken together, the biological findings verify the dose distribution of proton minibeams.

## 4. Discussion

In this study, we investigated the feasibility of pMBRT using an MSC and a scattering device consisting of multiple scatterers. The MSC was fabricated with a slit width and a leaf width of 2.5 mm. According to Sammer et al. [25], severe radiation toxicity was observed when the X-ray beam sizes were greater than 3 mm, but not when the beam sizes were less than 1 mm. If the clinical application of proton minibeam is considered, the slit width must be narrowed to reduce the radiation toxicity, the leaf width should be determined to achieve a high PVDR in the patient’s skin, and the PVDR must be actively modulated in the depth direction by multiple scatterers. The total thickness of the multiple scatterers can be determined by a look-up table that correlates a water depth satisfying PVDR = 1.1 with a scatterer thickness, and the table should be prepared on the basis of the measured dosimetric data. According to the film measurements, it can be seen that the width of SOBP was significantly reduced when only MSC was used without any scatterer (Figure 6). Because the proton minibeam has to treat the tumor in the same way as the broad beam, the conditions generating the minibeam, such as thickness of scatterer, norminal energy, and beam weights of SOBP, should be modulated. In this process, patient setup errors, range uncertainties, and proximal margins should be taken into account as well. PVDR is usually used as a measure to spare healthy tissues in pMBRT, but in this study, it was also used to examine the lateral dose homogeneity of the proton minibeams generated by a diverging MSC. This value has no information about the dose homogeneity in the depth direction, so the depth dose profiles should be checked (Figure 6).

The use of MSC provides some clinical advantages, including the reduction of proton beam penumbra and the improvement of PVDR by increasing the thickness of the MSC in the direction of beam propagation. It is well known that collimators reduce the penumbra of a spot proton beam [26]. Our study showed that increasing the thickness of the MSC results in an increase in the PVDR at the beam entrance. This is probably because the number of scattered particles passing through the MSC decreases as the MSC thickness increases. This is considered one of the key factors for the clinical application of proton minibeams.

However, the use of MSC also presents a few disadvantages, such as increased treatment time due to reduced output and the generation of secondary particles. For the MSC used in this study, the mean output in the SOBP region was approximately 50% that of the broad beam. This reduction in output increases the beam delivery time by approximately two times. If the slit width is narrowed to 1 mm, the beam delivery time is expected to increase by approximately five times compared to when a broad beam is used. Secondary particles are mainly generated by the scatterers and the MSC, and most of them are neutrons and low-energy electrons. Although the neutron dose is low in patients [27,28], it is desirable to reduce the neutrons, especially those with low energy (~1 MeV or less), because they have the highest relative biological effectiveness (RBE) and may increase the risk of secondary cancer in patients. For effective neutron shielding, a material containing a large amount of hydrogen and atoms with a large neutron absorption cross-section is required. Moreover, its water-equivalent thickness should be small to minimize the reduction in the range of the proton beam. It is expected that a polyethylene plate containing boron-10 could be used. Most secondary electrons with low energies can also be absorbed by the neutron shield.

Another way to generate proton minibeams with suppressed neutron production is to utilize a pencil beam scanning technique. This method has the advantage of being able to control the distance between the beam spots, which eliminates the necessity of patient-specific MSC. However, the method has the difficulties of reducing the spot size to 1 mm or less for all therapeutic proton energies at clinical proton therapy facilities because of the high beam emittance of medical circular accelerators [29] and the long source-to-axis distance in gantry systems [21,30]. As for the technique using MSC, a narrow proton beam with 1 mm or less is easily obtainable for all therapeutic proton energies. In addition, it is difficult to achieve a high PVDR near the skin while forming a uniform tumor dose, owing to the broad penumbra of the therapeutic proton pencil beam.

From our viewpoint, a more realistic strategy for the implementation of pMBRT at a clinical center is to maximize the PVDR in the patient’s skin using a well-designed MSC with sufficient thickness and then to modulate the PVDR in depth using scatterers. In the measurement of lateral dose profiles, a range shifter that reduces the minimum energy of the proton beam accelerated in proton accelerators (such as cyclotron, synchrocyclotron, and synchrotron) to 0 MeV was not considered separately but included in the PMMA phantom. This should be prepared for a more accurate evaluation of the pMBRT.

## 5. Conclusions

In this study, MC simulations and physical and biological experiments showed that the PVDR at the patient’s skin could be increased high enough to prevent radiation toxicity by an MSC with an appropriate leaf width and MSC thickness, and a uniform dose could be delivered to a tumor with large volume by a scattering device, even though the tumor was placed at a shallow depth. This means that pMBRT using an MSC and a scattering device is feasible in clinical proton therapy facilities. In this validation process, the factors affecting the performance of pMBRT, such as MSC-related factors and scatterer-related factors, were identified and better understood. The MSC-related factors included slit shape, leaf width, MSC thickness, and scatterer-related factors, including the thickness of scatterer and the air gap between the scatterer and the patient. Manipulating these factors may improve the efficacy of pMBRT in the treatment of tumors.

## Data Availability

Data presented in this study are available on request from the corresponding author.

## Supplementary Materials

The following supporting information can be downloaded at: https://www.mdpi.com/article/10.3390/cancers14122888/s1, Figure S1: Effect of leaf width of MSC on PVDR; Table S1: Energy and spot size of the proton pencil beam used in the measurement; Table S2: Measured lateral penumbra of proton minibeams generated by 10 cm thick MSC with leaf of 2.5 mm and c-t-c distance of 5 mm.
