# Supplementary material for: Dose Profile Modulation of Proton Minibeam for Clinical Application
Source: Cancers (Basel). 2022 Jun 11;14(12):2888. doi: 10.3390/cancers14122888 (PMC9221247; doi:10.3390/cancers14122888)
Supplement: Supplementary file 1 [file cancers-14-02888-s001.zip › cancers-1674334-supplementary.pdf]

Energy and Spot size of the proton pencil beam used in the measurement.

Table S1. Energy and Spot size of the proton pencil beam used in the measurement. The spot sizes (1 sigma) of the proton pencil beams were listed for each beam energy. The proton beams used in the experiment were highlighted in apricot. The lowest energy of the proton beam used to form 10 cm-wide SOBP was close to the minimum energy of our proton therapy system (Proteus-235, IBA, Louvain-la-Neuve, Belgium)

No.	Energy (MeV)	Spot size (1σ, cm)	
		X	Y
1	211.9	0.552	0.527
2	206.8	0.572	0.555
3	202.4	0.585	0.567
4	197.3	0.593	0.580
5	191.8	0.610	0.589
6	187.2	0.620	0.615
7	181.8	0.638	0.625
8	176.9	0.653	0.651
9	171.6	0.670	0.664
10	166.7	0.688	0.700
11	162.1	0.707	0.701
12	157.4	0.727	0.723
13	152.1	0.751	0.736
14	146.5	0.777	0.766
15	142.1	0.800	0.782
16	136.4	0.832	0.813
17	132.0	0.857	0.828
18	127.2	0.886	0.876
19	122.6	0.915	0.898
20	117.5	0.950	0.942
21	112.5	0.984	0.973
22	106.8	1.030	1.016
23	102.7	1.062	1.048
24	97.4	1.109	1.096
25	95.1	1.133	1.119

Delivered proton beams (SOBP=10 cm)

Effect of leaf width of MSC on PVDR

MC simulation was performed to investigate the effect of leaf width of a diverging MSC on PVDR.

From the simulation, the dose distribution with depth was plotted for the leaf widths of 0.6 mm, 1.0 mm, and 2.5 mm and c-t-c distances of 1 mm, 2 mm, and 5 mm, respectively (Figure S1). PVDR at the entrance increased as the leaf width increased, and it decreased with depth and converged to 1 for all MSCs. Higher PVDR is preferred to enhance the sparing effect around the skin, and it is achievable using a MSC with larger leaf width.

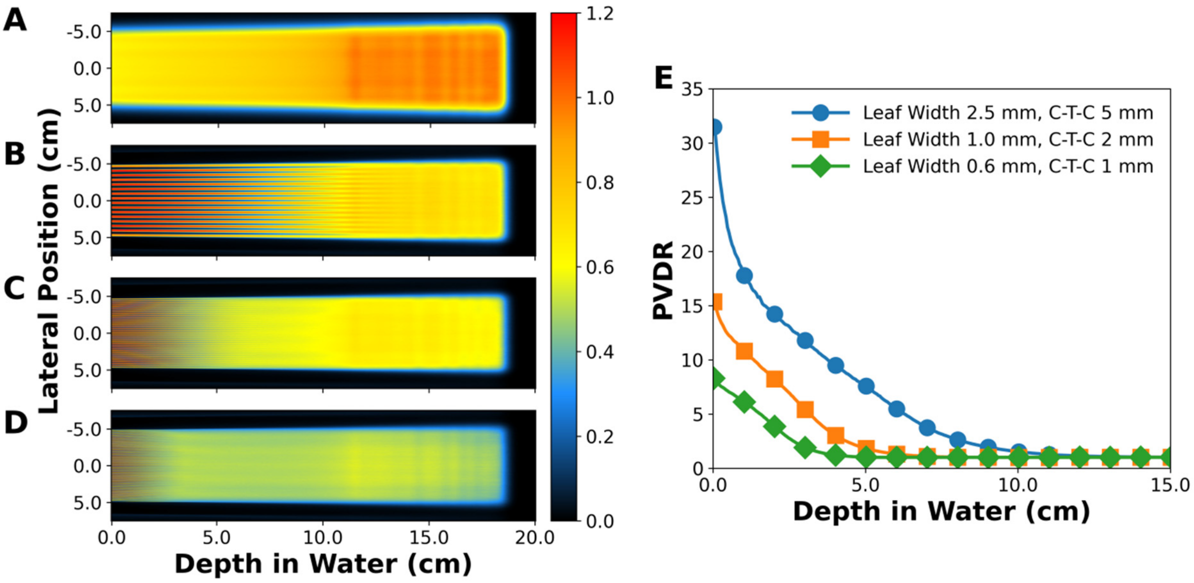

Figure S1. Effect of leaf width of MSC on PVDR. 2D-depth dose distributions obtained by MC simulations for (A) none-MSC, and the diverging MSCs with (B) 2.5 mm leaf width and 5 mm c-t-c distance, (C) 1 mm leaf width and 2 mm c-t-c distance, (D) 0.6 mm leaf width and 1 mm c-t-c distance, respectively, and (E) PVDR values with depth.

Effect of MSC and scatterer on lateral penumbra

The lateral penumbra of the proton broad beam can be changed by the MSC and the scatterer. The film measurements of the penumbra were summarized in Table S2. In principle, the lateral penumbra increases with depth due to multiple coulomb scattering. The table reflects this tendency well. As the MSC was applied, the penumbra dramatically reduced in all depths, and the scatterers increased the penumbra slightly.

Table S2. Measured lateral penumbra of proton minibeams generated by 10 cm-thick diverging MSC with leaf with of 2.5 mm and c-t-c distance of 5 mm.

		Depth (cm)	without a MSC		with a MSC					
			S_0		S_0		S_2		S_3	
			X	Y	X	Y	X	Y	X	Y
Surface		0.0	1.130	1.232	0.559	0.364	0.563	0.440	0.567	0.402
SOBP	Proximal	6.0	1.240	1.350	0.660	0.440	0.779	0.677	0.804	0.728
	Mid	12.0	1.278	1.380	0.775	0.614	0.927	0.851	1.020	0.931
	Distal	14.0	1.287	1.359	0.813	0.745	1.058	0.931	1.143	1.029

X=perpendicular direction to a long axis of slit, Y=parallel direction to a long axis of slit, MSC= multislit collimator, S_0 = without lead scatterer, S_2 = with 2 mm-thick lead scatterer, S_3 = with 3 mm-thick lead scatterer.
